# Bladder Perivascular Epithelioid Cell Neoplasm With Transcription Factor E3 Gene Rearrangement Treated by mTOR Inhibitor and Partial Cystectomy: A Case Report

**DOI:** 10.1002/iju5.70297

**Published:** 2026-11-05

**Authors:** Kimika Kaeriyama, Tadashi Kakitsuba, Wonseok Seo, Tetsuyuki Kurokawa, Akifumi Muramoto, Yuzuru Ariga, Koji Suzuki, Yoshiaki Imamura, Hideaki Okajima, Naoki Terada

**Affiliations:** ^1^ Department of Urology University of Fukui Fukui Japan; ^2^ Division of Diagnostic Pathology/Surgical Pathology University of Fukui Fukui Japan; ^3^ Department of Pediatrics University of Fukui Fukui Japan; ^4^ Department of Pediatric Surgery Kanazawa Medical University Kahoku Ishikawa Japan

**Keywords:** MTOR inhibitors, perivascular epithelioid cell neoplasms, transcription factor E3, urinary bladder neoplasms

## Abstract

**Introduction:**

Perivascular epithelioid cell neoplasms originating in the urinary bladder are rare, and their standard treatment methods have not been established.

**Case Presentation:**

A 14‐year‐old girl with intermittent macroscopic hematuria was referred to our hospital. Ultrasonography and magnetic resonance imaging revealed a 5 cm spherical mass in the bladder neck and transurethral biopsy revealed a perivascular epithelioid cell neoplasm. Chromosome G‐banding analysis indicated a transcription factor E3 gene rearrangement. The mTOR inhibitor, sirolimus, was administered for six months, followed by partial cystectomy. No tumor recurrence was observed after 15 months.

**Conclusion:**

This case suggests the potential efficacy of partial cystectomy combined with neoadjuvant mTOR inhibitor therapy for transcription factor E3 gene rearrangement‐positive perivascular epithelioid cell neoplasms.

## Keynote Message

Perivascular epithelioid cell neoplasms of the urinary bladder are rare, and their standard treatment methods have not been established. In this case of a 14‐year‐old girl, neoadjuvant mTOR inhibitor therapy successfully shrunk the tumor and clarified the surgical boundaries. This allowed for a bladder‐preserving partial cystectomy, achieving complete tumor resection while maintaining the patient's urinary function.

**Abbreviations**
CT: computed tomography
FDG‐PET: Fluorodeoxyglucose‐positron emission tomography
MRI: Magnetic resonance imaging
PEComas: Perivascular epithelioid cell neoplasms
TFE3: transcription factor E3
TSC: tuberous sclerosis complex

## 1 Introduction

Perivascular epithelioid cell neoplasms (PEComas) are a rare family of mesenchymal tumors composed of perivascular epithelioid cells that have features of both melanocytes and smooth muscles [1]. PEComas are characterized by mutations that lead to mTOR pathway activation, such as tuberous sclerosis complex (*TSC*) *1* or *TSC2* biallelic inactivation, transcription factor E3 (*TFE3*) gene fusion, and folliculin‐truncating mutations [2]. This case of PEComa harbored a *TFE3* gene rearrangement in a submucosal lesion of the urinary bladder. The patient was successfully treated with an mTOR inhibitor followed by partial cystectomy.

## 2 Case Report

A 14‐year‐old Japanese girl presented with intermittent macroscopic hematuria. Her height was 157 cm, and her weight was 53.8 kg. There were no significant findings in her medical history or physical examination. Ultrasonography at the previous medical facility revealed a 5 cm spherical mass in the bladder neck, and the patient was referred to our pediatric department. Blood tests and investigations related to tumor markers and endocrine function revealed no significant abnormalities. Magnetic resonance imaging (MRI) showed a 5 cm submucosal tumor in the bladder neck, while contrast‐enhanced computed tomography (CT) demonstrated a highly vascular mass in the same region. Fluorodeoxyglucose‐positron emission tomography (FDG‐PET) revealed positive tumor uptake (Figure 1). The I‐123 metaiodobenzylguanidine scintigraphy showed no significant uptake, indicating that the tumor was not a pheochromocytoma.

**FIGURE 1:**
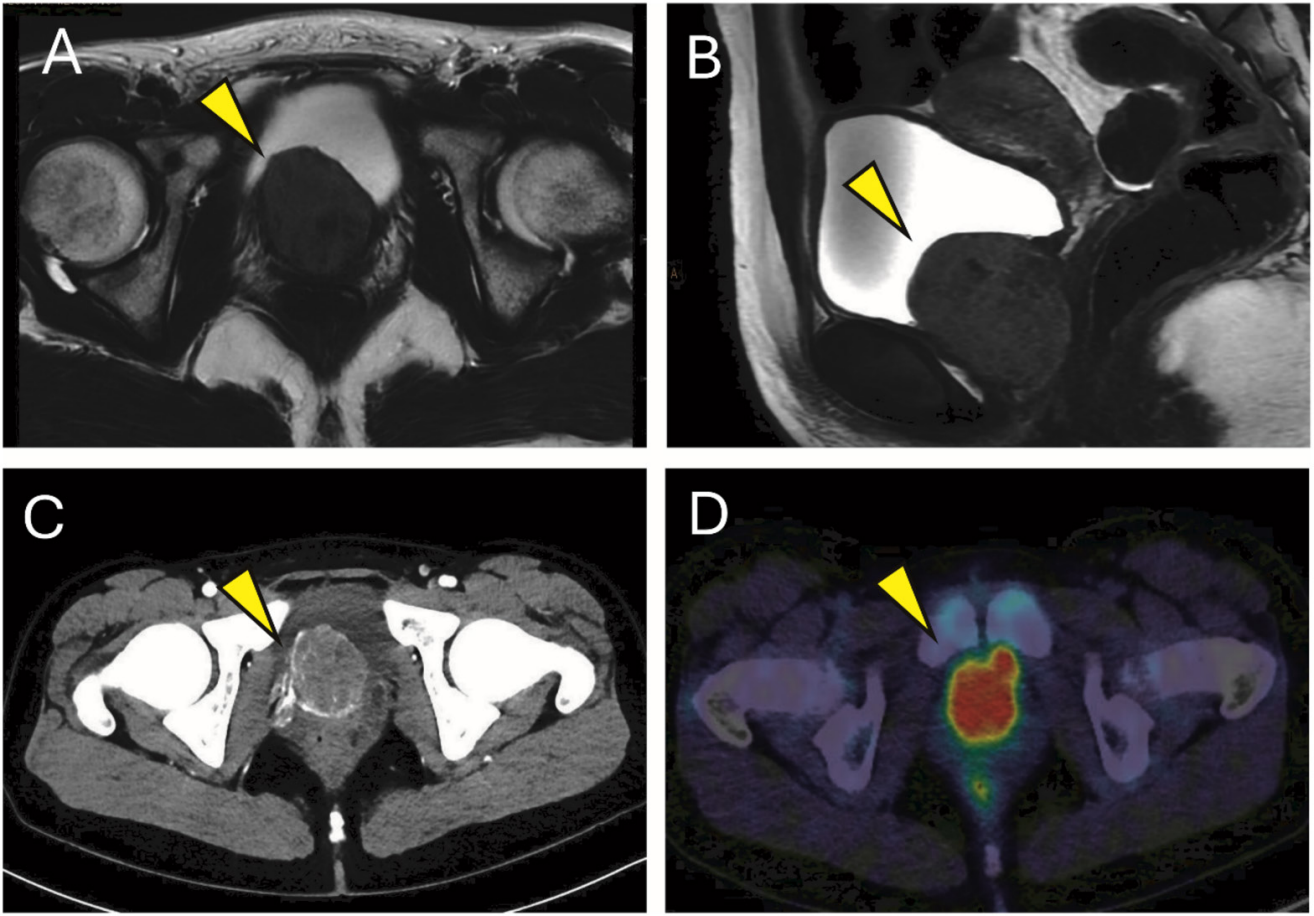
Initial imaging examinations of the 14‐year‐old female patient. (A, B) MRI. The arrows indicate the bladder mass (50 × 38 × 38 mm). (C) Contrast‐enhanced CT demonstrating a branch arising from the right internal iliac artery. (D) FDG‐PET showed high uptake in the tumor. Arrows indicate the tumor.

The bladder tumor was subjected to transurethral resection. Pathological examination revealed both tumor cells and stromal components. The tumor cells exhibited round to slightly irregular nuclei and poor differentiation. The tumor predominantly consisted of atypical cells with a high nuclear‐to‐cytoplasmic ratio, surrounded by a fibrous vascular stroma consisting of spindle‐shaped cells. Immunohistochemical staining revealed that the tumor cells were positive for desmin, HMB45, and TFE3, but negative for S‐100, SOX10, Melan A, CK AE1/AE3, calponin, myoglobin, CD56, and DOG1. Cathepsin K is also negative in this case. The fibrovascular stroma interspersed between the tumor cells was positive for HHF‐35, SMA, and CD34. The Ki‐67 labeling index was approximately 20% (Figure 2). These findings are consistent with a diagnosis of PEComa [3]. Chromosome G‐banding analysis using tumor tissue revealed a karyotype of 46,X,t (X;1) (p11.2;p36.1), indicating translocation and the possibility of a *TFE3* abnormality (Figure 3).

**FIGURE 2:**
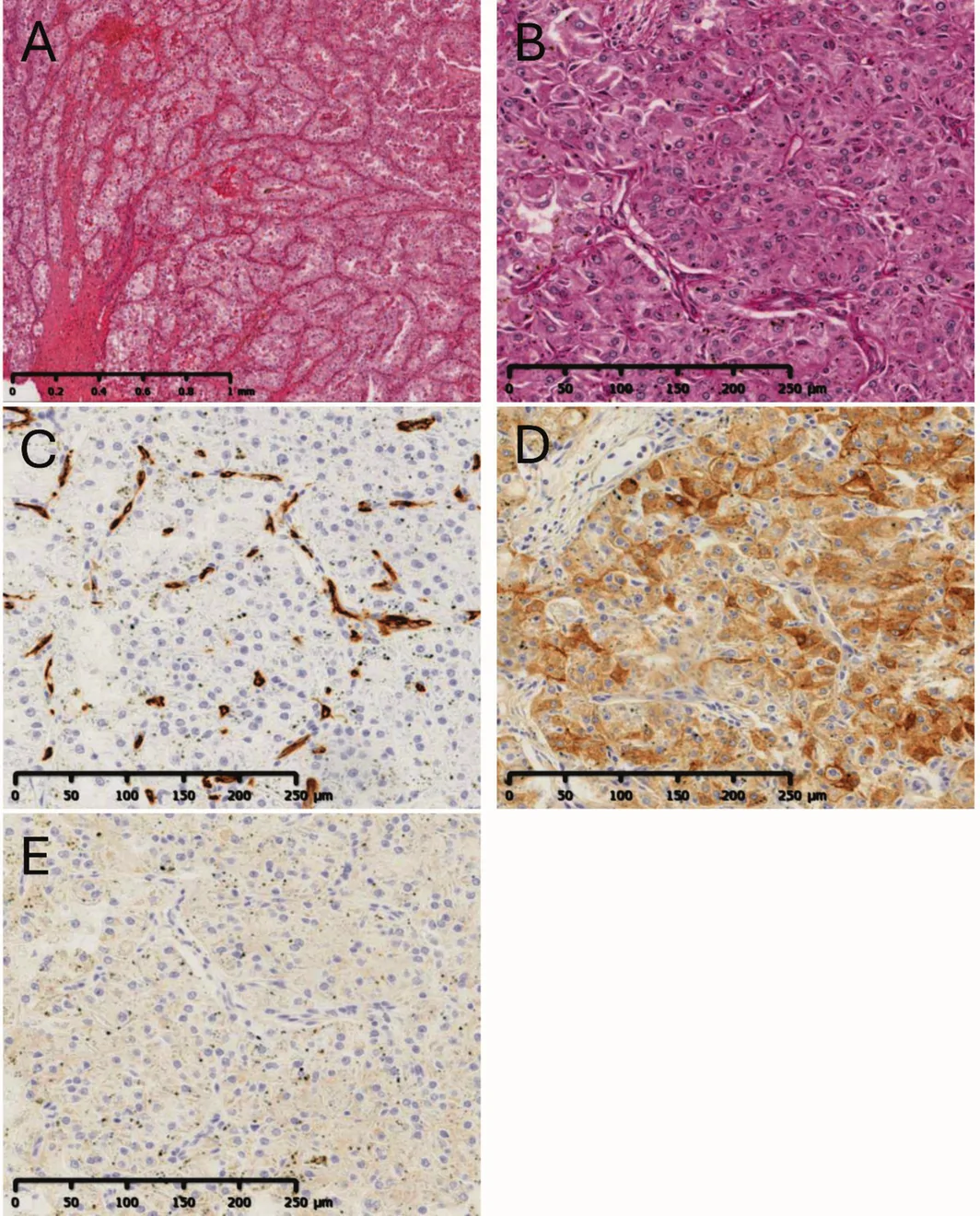
Histopathological findings. (A) Hematoxylin and eosin staining showing nests, papillary, and tubular proliferations of polygonal cells with abundant eosinophilic cytoplasm. (B) Periodic acid–Schiff staining reveals no intracytoplasmic granules. (C) Immunohistochemical staining for CD34 is positive in intervening vessels. (D) HMB‐45 is positive in the cytoplasm. (E) Cathepsin K is negative.

**FIGURE 3:**
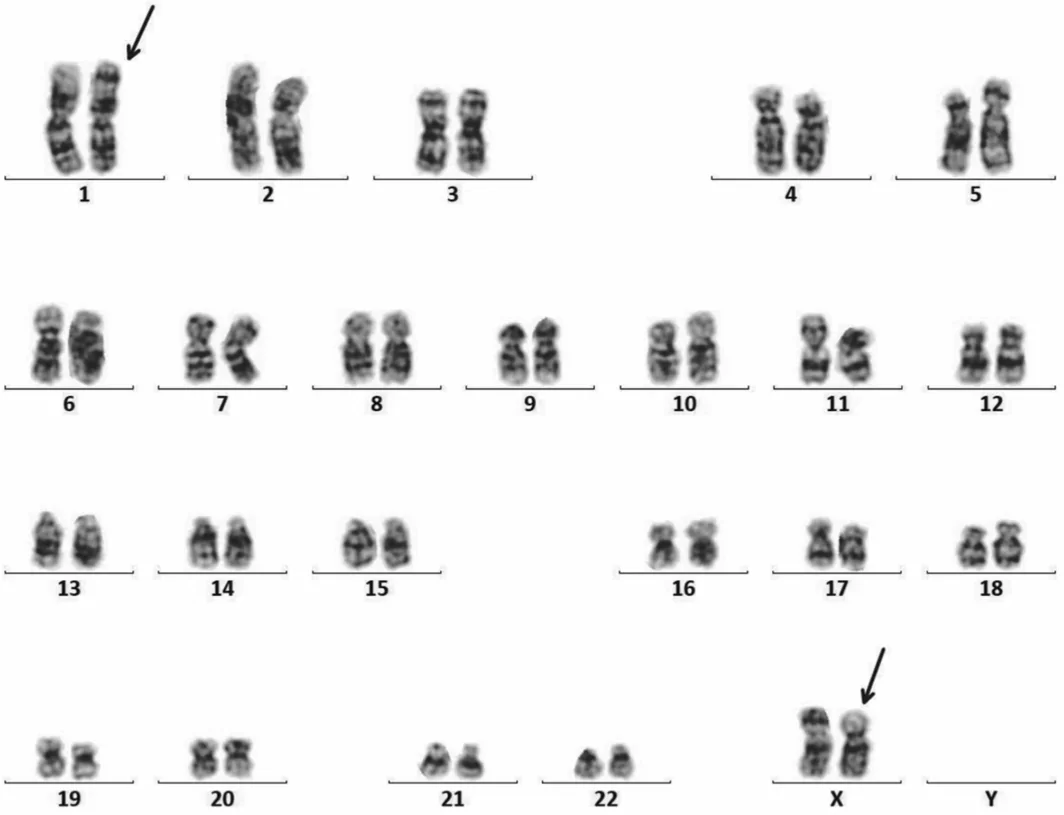
Chromosome G‐banding analysis showing a karyotype of 46,X,t (X;1) (p11.2;p36.1). Arrows indicate the translocation.

As the tumor was located in the bladder neck, radical cystourethrectomy and an ileal conduit were required to completely remove all tumor tissues. However, considering the patient's age of 14 years and the low malignant potential of the PEComa, we aimed to preserve the bladder. Reports of tumor shrinkage with mTOR inhibitor therapy in uterine PEComa cases led us to consider mTOR inhibitors based therapy in this case [4]. The oral mTOR inhibitor sirolimus was initiated at a dose of 4 mg/day (2.59 mg/m^2^). The dosage was subsequently adjusted to maintain whole‐blood trough levels within the target range of 5–15 ng/mL. After 6 months of treatment, follow‐up evaluations with ultrasound and MRI showed a clearer demarcation between the tumor and the bladder wall, as well as the formation of a stalk not observed at the initial presentation (Figure 4). Although the overall tumor dimensions showed a slight change from 36 × 44 × 48 mm (volume: approximately 40.0 cm^3^) to 49 × 28 × 46 mm (volume: approximately 33.0 cm^3^), this morphological deformation and stalk development made tumor enucleation feasible, and a partial cystectomy was performed. Under a transverse incision in the lower abdomen, the bladder wall was incised, and the tumor was identified at the bladder neck. The feeding arteries were ligated, the tumor was completely resected, and defects in the bladder mucosa and muscle were sutured. Owing to the hypervascular nature of the tumor and the requirement for meticulous dissection and hemostasis to avoid thermal injury to the nearby bilateral ureteral and internal urethral orifices, the total operative time was 373 min, with a loss of 1800 mL blood. Postoperative CT at 15 months showed no signs of recurrence and the patient had no urinary symptoms (Figure 4).

**FIGURE 4:**
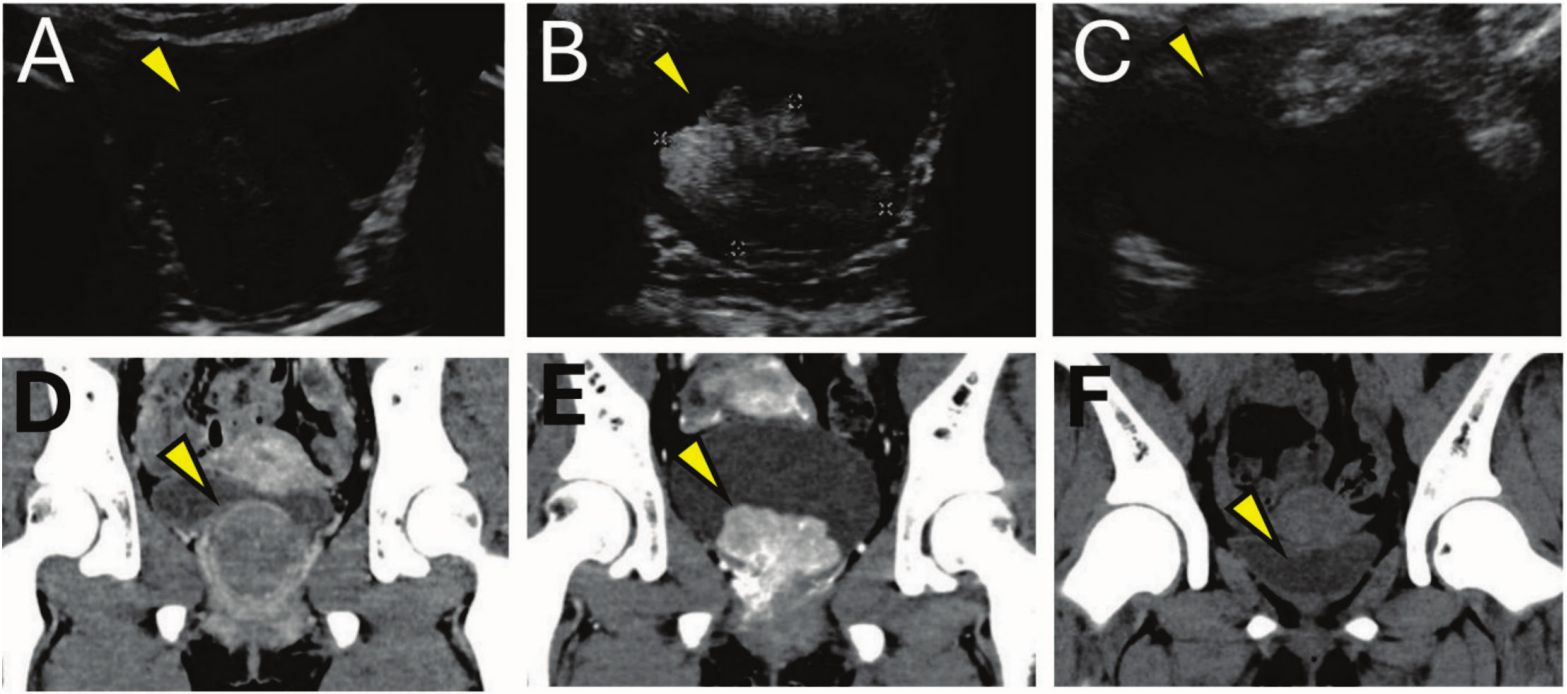
Serial imaging findings. (A–C) Ultrasonography (US) images: (A) Before initiation of mTOR inhibitor therapy, (B) 2 months after the treatment, and (C) 15 months postoperatively with no evidence of recurrence. (D–F) Computed tomography (CT) images: (D) At initial diagnosis, (E) 3 months after the treatment, and (F) 15 months postoperatively with no evidence of recurrence. Arrows indicate the tumor.

## 3 Discussion

PEComas have been documented in various anatomical locations such as the liver, kidneys, lungs, and uterus. However, only 36 cases of bladder PEComas have been reported so far, making it extremely rare [5]. As a result, there is no consensus on the treatment of bladder PEComa. However, surgical resection is considered the first‐line option, and partial cystectomy was performed in this case.

PEComas are commonly associated with tuberous sclerosis. Upregulation of mTOR signaling due to the inactivation of *TSC1/2* is considered a key oncogenic driver in this disease. Recently, a subgroup of PEComas harboring *TFE3* rearrangements and presenting a distinctive morphology was identified, and *TSC1/2* and *TFE3* alterations are mutually exclusive [6]. Notably, mTOR inhibitors inactivate TSC1/2 to target the upregulation of mTOR signaling. A comparative study of medical treatments by Sanfilippo et al. revealed that mTOR inhibitors were the most effective agents for PEComa [7]. Currently in Japan, the use of mTOR inhibitors for PEComa remains off‐label and is not covered by national health insurance. Therefore, implementation of this therapy requires careful ethical consideration; in the present case, the treatment was initiated after a thorough explanation of the potential risks and benefits was provided, and fully informed consent was obtained from the patient and her parents. However, due to this mutual exclusivity, TFE3‐positive PEComas are generally considered to exhibit a less robust response to mTOR inhibitors than TSC‐mutated PEComas. Nevertheless, this traditional dichotomy has been challenged by recent clinical evidence. For instance, cases of TFE3‐associated PEComa achieving a complete response (CR) to mTOR inhibitor therapy have been documented [5]. Furthermore, emerging molecular studies have identified rare malignant PEComas harboring both TSC1 alterations and TFE3 activation, indicating a more complex and overlapping oncogenic landscape than previously recognized [8].

Lin Yin Ong et al. reported that while preoperative chemotherapy did not necessarily lead to a reduction in tumor size, it significantly decreased tumor vascularity in the case of pediatric vaginal PEComa [8]. Our patient harbored a TFE3 rearrangement‐associated PEComa with Xp11 translocation, in which the tumor stalk became apparent following treatment with an mTOR inhibitor, allowing complete removal of the tumor tissue with enucleation surgery alone. To the best of our knowledge, this is the first report to demonstrate that preoperative mTOR inhibitor therapy for TFE3 rearrangement‐associated PEComa resulted in significant tumor deformation, facilitating successful surgical management.

A limitation of this report is that direct molecular confirmation—such as break‐apart fluorescence in situ hybridization, reverse transcription‐polymerase chain reaction, or RNA‐based next‐generation sequencing—was not performed. Nevertheless, the diagnosis of a TFE3 rearrangement‐associated PEComa was strongly supported by the characteristic G‐banding karyotype [46,X,t (X;1) (p11.2;p36.1)] and nuclear TFE3 expression on immunohistochemistry.

Postoperative CT at 15 months showed no signs of recurrence and the patient had no urinary symptoms. This represents a minimally invasive approach, which is particularly beneficial in pediatric patients and may enhance the future quality of life.

## Consent

Informed consent was obtained from the patient.

## Conflicts of Interest

The authors declare no conflicts of interest.

## Data Availability

The data presented in this study are available on request from the corresponding author. The data are not publicly available due to privacy or ethical restrictions.
